# A Verbal De-escalation Standardized Patient Workshop for Third- and Fourth-Year Medical Students

**DOI:** 10.15766/mep_2374-8265.11417

**Published:** 2024-07-19

**Authors:** Neeta Shenai, Valerie Fulmer, Catherine Gowl, Jordan See, Ryan Peterson, Reed Van Deusen

**Affiliations:** 1 Associate Professor of Psychiatry, University of Wisconsin School of Medicine and Public Health; 2 Director of Standardized Patient Program, University of Pittsburgh School of Medicine; 3 Standardized Patient Program Manager, University of Pittsburgh School of Medicine; 4 Assistant Professor of Medicine, University of Pittsburgh School of Medicine; 5 Assistant Professor of Psychiatry, University of Pittsburgh School of Medicine; 6 Associate Professor of Medicine, University of Pittsburgh School of Medicine

**Keywords:** Patient Agitation, Verbal De-escalation, Internal Medicine, Physician-Patient Relationship, Psychiatry, Standardized Patient

## Abstract

**Introduction:**

Verbal de-escalation is an essential skill for physicians across specialties and is the first-line intervention for patients who present with agitation. Training in verbal de-escalation for medical students is less robust compared to other health care disciplines. We describe the creation and evaluation of a novel verbal de-escalation curriculum for third- and fourth-year medical students on their psychiatry clerkship rotation.

**Method:**

We developed a simulation using standardized patient (SP) methodology and a dedicated reflection session, implementing it in the third-year psychiatry clerkship. Participants in the scenario received targeted feedback from their peers and SPs. The sessions were video recorded, and a random sample was selected and reviewed to identify key observations and themes from student performance.

**Results:**

A total of 139 students participated in the encounter. One hundred twenty-two of 125 students (82%) stated the activity met the learning objectives, with 108 (86%) assigning the letter grade A to the activity. Written feedback indicated that the majority of students believed the activity to be realistic, instructive, and helpful but felt the SPs de-escalated too quickly. Video review of the encounters found that while the students effectively used the skills, many jumped to a quick fix, and some offered inappropriate choices to end the encounter.

**Discussion:**

This SP activity was effective in allowing students to practice skills in a safe setting and was valued by students. In the future, adding another workshop in the fourth year could facilitate higher retention and practice of skills.

## Educational Objectives

By the end of this activity, learners will be able to:

1. Apply the techniques of verbal de-escalation to a realistic simulated scenario with a standardized patient displaying anger and agitation.
2. Identify one element of the encounter that went well and one element that could be improved through a student-led reflection debrief.

## Introduction

Patients admitted to the hospital often feel tremendous anger, frustration, fear, and stress. These strong emotions can escalate to agitation, aggression, and violence, which threaten the health and safety of patients and hospital staff. A 2019 meta-analysis showed that over 60% of health care workers reported exposure to some form of workplace violence and 57% reported verbal abuse in the hospital.^1^ The Occupational Safety and Health Administration reports that up to 75% of all workplace assaults occur in hospitals and health care settings.^2^ The highest prevalence is often found in the emergency department and psychiatry units, with almost 20% of patients admitted to acute psychiatric wards committing some act of violence during their admission.^3^ Since the COVID-19 pandemic, global rates of workplace violence have only risen in health care settings, likely reflecting stress and uncertainty during already difficult times.^4^

For providers, these events can have significant negative social and psychological consequences. Verbal and physical assault from patients can lead to post-traumatic stress disorder and anxiety as well as decreased job satisfaction and burnout.^5,6^ Patient outcomes are often negatively affected as violence leads to chemical sedation, physical restraint, and refusal of care that frequently increase length of stay.^7^ For these reasons, acute patient agitation is considered a behavioral health emergency, with verbal de-escalation as a first-line intervention.^8^ There is clear value in implementing skills to de-escalate patient agitation and prophylactically mitigate conflict. One study found that developing an interdisciplinary team-based de-escalation curriculum for emergency department staff helped to reduce safety events.^9^

While many training programs currently exist for mental health providers, nurses, and physicians,^10–13^ comprehensive verbal de-escalation training in undergraduate medical education is much less robust. A video series on verbal de-escalation skills increased medical student comfort and confidence in management of agitated patients, and students found this educational content valuable.^14^ Interpersonal communication is a well-accepted pillar in medical school curricula. The Association of American Medical Colleges has identified patient-centered interviewing skills as its first Entrustable Professional Activity, with particular attention to verbal and nonverbal cues and active listening.^15^ The advanced interviewing skills needed to engage an agitated patient, establish a therapeutic relationship, and de-escalate the situation should be routinely taught for a comprehensive medical education.

At the University of Pittsburgh School of Medicine, medical students develop their interpersonal communication and history-taking skills through a longitudinal curriculum with the use of standardized patient (SP) methodology. Experiential learning through simulated case scenarios with an SP is then debriefed to encourage retention. While students are exposed to advanced interviewing skills in the second year, the technique of verbal de-escalation has not specifically been taught in the formal curriculum. We describe a novel program using SP practice and a dedicated reflection session intended for third- and fourth-year students on their psychiatry clerkship rotation. The program has been designed to help participants recognize the evidence-based skills of de-escalation, apply them to a simulated encounter, and reflect on their performance through a student-led debrief.

## Methods

### Case Design

An interdisciplinary team of faculty representing internal medicine and psychiatry worked in conjunction with the University of Pittsburgh's SP program to create two simulated cases. While the activity was included as part of the psychiatry clerkship curriculum, the cases in the clinical scenarios purposefully did not have primary psychiatric diagnoses in order to emphasize the universality of the application of de-escalation skills. We intentionally developed each case to have different types of agitation, given that agitation exists on a continuum.^16^ One case focused on a patient in the inpatient setting who presented with endocarditis and wished to leave against medical advice. This case highlighted explosive anger, with a patient actively throwing clothes into their bag and using a loud tone of voice, profanity, big gestures, pacing, and purposeless movements. The second case was set in an outpatient primary care clinic where the patient was inappropriately demanding controlled substances to manage their insomnia. This patient displayed agitation through passive-aggressive language, belittling, irritability, poor eye contact, frequent interruptions, rude behaviors, and a sense of entitlement. The disparity between these phenotypes of agitation allowed the student to practice different techniques of verbal de-escalation and also emphasized how these techniques were essential in all clinical settings. Both cases were modeled after real patient scenarios that were common examples of agitation encountered in the two settings (Appendices A and B).

### SP Preparation

SP training was an iterative process. The materials were piloted by role-play with two training team SPs, medical students, course directors, and SP administration for 4 hours, after which calibration of the materials and SP instructions occurred. Eleven SPs attended a 4-hour training, where we discussed the objectives of each case and the unique qualities of the portrayals. Levels of escalation were role-played in addition to variables such as the varying ranges of student skills that would impact escalation and de-escalation and the language appropriate in each case (Appendix C).

### Patient Encounter

Students were given an article^8^ ahead of time to review the core techniques of verbal de-escalation. On the morning of the activity, students received a 20-minute didactic primer on verbal de-escalation techniques from the clerkship director (Appendix D). The structure and goals of the session were reviewed at this time to emphasize that the goal was not necessarily to find a solution to the presenting problem but rather to practice and apply verbal de-escalation skills to mitigate agitation. Students were also encouraged to take a time-out during the case scenario should they need one. At the University of Pittsburgh, all medical students had been exposed to the time-out concept from their first year. At any point while interacting with an SP, a student could call a time-out if they got stuck during an encounter, and the SP would stop interacting to allow the student the opportunity to discuss directly with the facilitator the best path forward. In our encounter, the time-out would pause the scenario and allow the student to confer with their partner on what to do next.

Four separate simulation rooms ran simultaneously: two rooms for the outpatient case and two rooms for the inpatient case. The clerkship coordinator was present in person to ensure the cases were running on time. During the activity, students were divided into groups of two, and each student had an opportunity to interview one case and observe another. Each case was 22 minutes in length: 1 minute to read the case prompt (Appendices E and F), 12 minutes to interview the SP, 2 minutes for the SP to fill out the feedback form (Appendix G), and 7 minutes for debriefing with the SP (Appendix H). The total duration of the activity was approximately 2.5 hours.

### Session Debrief

While no prior training was offered, the observing student was given specific instructions for leading the debrief along with a printed script. If the interviewing student called a time-out, the observing student used the script to lead the discussion with questions such as “What caused you to call time-out?”, “What do you want to achieve when you time back into the encounter?”, and “What words will you use to accomplish that goal?” At the end of the session, the observing student first asked the student and then asked the SP, “What do you think went well?” This was followed by the observing student asking the student and then the SP, “What could be improved?” This prompted the interviewing student to reflect on the scenario and included the SP for feedback on areas for growth (Appendix H). During the debrief, SPs were permitted to step out of their role and offer feedback. SPs used a feedback form outlining the verbal de-escalation techniques to help facilitate specific formative feedback (Appendix G). At the end of the session, students received a handout outlining the core verbal de-escalation techniques to reinforce the concepts (Appendix I). The clerkship director was available as needed if a student requested additional debriefing; however, no students elected to use this resource.

### Learner Assessment

Following the activity, students were given an evaluation form that asked them to rate the overall quality of the session and provide feedback on areas of strength and areas for growth (Appendix J). To evaluate the quality of the encounters and to identify common themes from the students, video review was used. Twelve video-recorded encounters were randomly chosen and provided to two members of the team. Specific elements of the sessions were identified, including each encounter's length and whether the student used a time-out. The SP's level of agitation was documented, with a particular focus on how quickly into the encounter they de-escalated their emotion. Finally, common responses and behaviors from the students were documented, along with feedback for SP improvement in future iterations (Table).

**Table.**
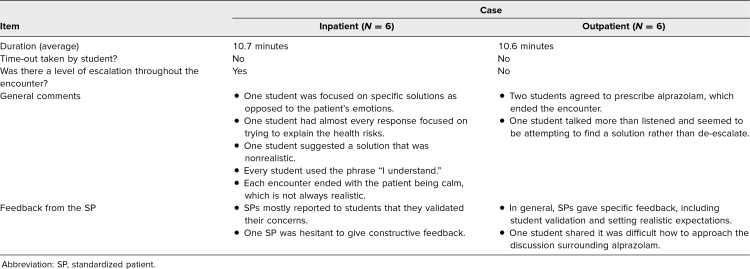
Results of Video Review of 12 Random Encounters

This activity was exempted from institutional review board oversight, as determined by the Research on Medical Students Review Committee of the University of Pittsburgh School of Medicine.

## Results

### Student Evaluation

The activity was run every 4 weeks with each new cohort of psychiatry clerkship students. A total of 139 students participated. The session took place 12 times over 12 consecutive months. One hundred twenty-five students submitted feedback for the exercise. On a 5-item Likert scale ranging from *strongly agree* to *strongly disagree,* 102 of 125 students (82%) strongly agreed, 20 (16%) agreed, two (2%) were neutral, and one (1%) disagreed that the workshop met the stated learning objectives. One hundred eight students (86%) assigned the letter grade A for educational quality, 16 (13%) rated the quality a B, and one student (1%) rated the quality a C.

### SP Encounter Observations

A random sample of 12 video-recorded encounters was reviewed. Both types of cases ended on time on average, with the outpatient case lasting an average of 10.6 minutes and the inpatient case lasting 10.7 minutes. For all sessions reviewed, no student utilized a time-out. The SPs maintained their level of agitation throughout the encounter during the inpatient case, but in the outpatient case, their level of agitation dropped much quicker. In general, students commonly jumped straight to fixing the problem before utilizing any other de-escalation technique. On multiple occasions during the encounter where the patient was requesting a controlled substance to sleep, the students inappropriately promised them alprazolam, which led to the encounter ending prematurely.

### SP Feedback

Separate debriefing sessions were held with the SPs at a later date to glean their thoughts on improving the quality and clarity of instruction in the cases. They reported difficulty with maintaining a high level of agitation throughout the case and were confused on the timing at which they should appropriately begin to reward the students’ skill and de-escalate their emotion. Several SPs reported surprise at the students’ conceding they could have everything they were asking for, even if the medication was medically inappropriate, and did not know how to proceed with the case from there.

### Participant Feedback

In their narrative feedback regarding the best aspects of the workshop, students highlighted the relevance to real life de-escalation situations:

• “Having a realistic situation… forced you to react fast to de-escalate.”
• “It was helpful practicing these stressful situations to have some idea how to handle them in the real world.”

Students also cited feedback from the SPs and support from having a fellow learner observe the activity as strengths:

• “The feedback was very valuable.”
• “Real time feedback.”
• “I also really liked that we got to serve as observers for our fellow medical students. It felt a bit like having a supporter in your corner while in the room.”
• “To go in with another student to practice. Helps to have someone to bounce ideas off of and to help relieve some of the stress.”
• “I thought the feedback was useful. It all felt like a more useful instructional experience than prior SP sessions. The SPs themselves were great at inhabiting their personas as usual.”
• “I enjoyed the opportunity to practice difficult conversations and de-escalation tactics in a controlled environment. This was a very valuable learning opportunity.”

Improvements recommended by the students focused on more preparation and demonstration of de-escalation techniques prior to the simulation. Written feedback on areas of growth centered on obtaining more opportunities for practice.

• “We were in pairs for inpatient/outpatient de-escalations but I would prefer getting a chance to practice both interactions rather than one.”
• “My SP ‘deescalated’ a bit quickly—not sure if that was because the conversation was going well or not. Maybe more time to practice dealing with a hostile patient.”

## Discussion

To our knowledge, this is the first SP training for medical students in verbal de-escalation. Written feedback from students participating in the workshop demonstrated that they valued the training and rated its quality as high. Based on this feedback, we believe that utilizing SP methodology is an effective modality for medical students to apply the techniques of verbal de-escalation and have an opportunity to reflect on the encounter.

Though simulation-based training is more efficacious,^10^ implementation of a case scenario of an agitated patient is challenging, especially for trainees. An important lesson was balancing the SP's intensity of escalation with the student's skill level. We discovered that ending the encounter when the patient was still agitated was uncomfortable for both the student and the SP, as well as potentially distressing. We discussed during the didactic primer that it was not uncommon to feel the encounter was not satisfactory even after verbal de-escalation techniques had been applied successfully. We instructed the SPs to maintain escalation while the student practiced two verbal de-escalation interventions; while this helped decrease student discomfort, some students felt the SP de-escalated too quickly. In contrast, while a minority of students felt distressed after the activity, none utilized the option of an additional one-on-one debrief with the clerkship director.

In video review of the sessions, we found that although students were encouraged to take a time-out, the majority of them did not. We also learned that, though students were advised prior to the session that the goal of the encounter was to practice verbal de-escalation techniques, a cohort focused on fixing the patient's problem. When this occurred, it made measuring the effectiveness of the learning activity in achieving the educational objectives challenging. While at times verbal de-escalation and problem-solving might be linked, in a minority of students this led to premature offering of alprazolam for sleep in the outpatient case, which was not an accurate clinical recommendation and effectively ended the encounter. The offer quickly and successfully de-escalated the patient but in a manner that was not clinically appropriate. This emphasizes how the approach of verbal de-escalation is distinct from other forms of communication in medicine and how students may benefit from a more comprehensive didactic primer prior to the activity, as well as additional simulated practice.

As a result of the feedback, we revised the SPs’ training and case instruction to help them calibrate and maintain a level of escalation. Specifically, we included more detailed responses on how the SP could maintain escalation in the encounter and added a sample script. Additionally, we provided instructions to the SPs that in an escalated state, it should sometimes take two or three times to process behaviors and statements made by the learner before the SP de-escalated.

We found that rotating SPs to have no more than two to three student encounters in a row was essential to the maintenance of emotional safety and quality of role portrayal. In the inpatient case, instruction was given to reescalate with the desire to smoke for each encounter, as initially this was not completed consistently. For the outpatient case, if the student agreed to prescribe alprazolam, the SP provided feedback to the student that the medication was not an ideal choice and to discuss further with the clerkship director for additional clarification. Finally, we added content to the didactic primer to emphasize the goals of the session.

There were specific limitations associated with this study. In the activity, students knew they were entering a situation that required use of verbal de-escalation skills, which may not mimic a real-world situation. While the students were exposed to both simulated cases, each student was able to practice only the inpatient or outpatient case. There was also no formal assessment of the students’ use of and skill with the various verbal de-escalation techniques.

Verbal de-escalation of an agitated patient is an essential skill for physicians across specialties. In the future, a practical extension of this curriculum would be to include training to identify the signs and symptoms of early agitation so as to prevent escalation of the situation. We also plan to add an advanced SP workshop on verbal de-escalation in the fourth year to allow more opportunities to practice in more complex situations, such as encounters with family members. An assessment component with a postcurriculum OSCE could also be developed to assess skill acquisition. As gaining mastery of these skills is challenging, inclusion of a verbal de-escalation curriculum across all medical schools would bridge this gap.

## Appendices


SP Cases.docxLogistics.docxWorkshop.docxVerbal De-escalation Primer.pptxCase 1 Prompt.docxCase 2 Prompt.docxSP Learner Feedback.docxInstructions for Observing Learner-Led Debrief.docxStudent Handout.docxStudent Evaluation Form.docx
*All appendices are peer reviewed as integral parts of the Original Publication.*
