# Supplementary material for: A Verbal De-escalation Standardized Patient Workshop for Third- and Fourth-Year Medical Students
Source: MedEdPORTAL. 2024 Jul 19;20:11417. doi: 10.15766/mep_2374-8265.11417 (PMC11258212; doi:10.15766/mep_2374-8265.11417)
Supplement: Supplementary file 1 — SP Cases.docxLogistics.docxWorkshop.docxVerbal De-escalation Primer.pptxCase 1 Prompt.docxCase 2 Prompt.docxSP Learner Feedback.docxInstructions for Observing Learner-Led Debrief.docxStudent Handout.docxStudent Evaluation Form.docx [file mep_2374-8265.11417-s001.zip › B. Logistics.docx]

**Appendix B: Logistics**

1. Location of Activity
   1. University of Pittsburgh School of Medicine small group rooms
2. Setting for the Encounter
   1. Primary care office for outpatient case
   2. Inpatient medical hospital for inpatient case
3. Materials Required
   1. Props in room
      1. Examination table
      2. IV pole
      3. Hospital gown
      4. Bag with clothes
      5. Cell phone
4. Administrator Packet
   1. Schedule: include student names, SP names, room number assigned for each group of students
   2. Student instructions for the activity
   3. Student post-activity hand-out
   4. Feedback rubric for SP to complete for each student
5. Number of encounters completed by each learner
   1. For this activity, each student learner completed two standardized patient (SP) encounters and served as the interviewer and observer for one case.
6. Length of each encounter
   1. Students were given 1 minute to read the case prompt and 12 minutes to interview
   2. SP’s were given 2 minutes to fill out the SP facilitator checklist
   3. Students were given 7 minutes to de-brief with the SP.
   4. After the activity, students were given a hand-out with review of verbal de-escalation techniques and available resources for independent learning
   5. After completing the activity, students were invited to complete an online evaluation
7. Total number of encounters expected in a session for the SP
   1. SPs had two cases a day
8. Start and finish time
   1. 44 minutes per group
9. Staffing Requirement: 4 SP rooms run simultaneously
   1. 1 standardized patient needed per room per day
   2. A minimum of one administrator per day
10. Pre-briefing material given to learners prior to attending session
    1. Reading and resources for verbal de-escalation provided to learners in advance (see Appendix I and article)
11. Briefing orientation materials used
    1. Student instructions and primer didactic for verbal de-escalation for the session are provided in Appendix D
12. Describe feedback and or debriefing methods used
    1. A SP facilitator feedback form is found in Appendix G
    2. A guide for the learner led de-brief is found in Appendix H
13. Feedback provided post-activity to learners
    1. None
