# Supplementary material for: A Verbal De-escalation Standardized Patient Workshop for Third- and Fourth-Year Medical Students
Source: MedEdPORTAL. 2024 Jul 19;20:11417. doi: 10.15766/mep_2374-8265.11417 (PMC11258212; doi:10.15766/mep_2374-8265.11417)
Supplement: Supplementary file 1 — SP Cases.docxLogistics.docxWorkshop.docxVerbal De-escalation Primer.pptxCase 1 Prompt.docxCase 2 Prompt.docxSP Learner Feedback.docxInstructions for Observing Learner-Led Debrief.docxStudent Handout.docxStudent Evaluation Form.docx [file mep_2374-8265.11417-s001.zip › C. Workshop.docx]

**Appendix C. VERBAL DE-ESCALATION STANDARDIZED PATIENT WORKSHOP**

**Structure Overview of Each Case:**

Format: very small group unfacilitated. Two learners, one SP.

1 minutes: Observing student reads prompt

2-13 minutes: Student 1 interviews (student 2 observes, interviews in other room).

14-20 minutes: Debrief.

**Clinical Scenario 1:** [INPATIENT] 20 minutes

Observing student reads this prompt to the interviewing student: You were notified by your intern that Taylor is threatening to leave the hospital AMA. As the intern is in a code, the intern requested that you assess the needs of the patient until the intern is available because the patient is packing up their stuff to leave right now. The intern tells you that this would be a high-risk situation for the patient to leave AMA given the severity of their endocarditis. The patient needs six weeks of IV antibiotic treatment and given prior history of OUD (stable now), currently there is no other disposition option except inpatient hospitalization. To add to the complexity, the intern tells you that per hospital rules, the patient is not allowed to leave the unit due to a history of OUD and current PICC line.

Case Overview:

Taylor Jackson is a 20-55 y.o. person who presented to the medical hospital six days ago for evaluation of chest pain. Initial history and workup reveals a diagnosis of endocarditis on TTE with a small vegetation on the aortic valve. Taylor has a remote history of opioid use disorder and has been stable for many years.

PMHx: none

Past psychiatric Hx: unknown

Substance Use Disorder history:

Nicotine: 1 ppd for 20 years

Opioids: history of opioid use disorder and has been stable for many years on methadone

Alcohol use: denies

Crack/cocaine: denies

Cannabis: yes, reports exact amount is hard to quantify; “a little bit here and there”

Amphetamines: denies

Hallucinogens: denies

Allergies: none

Current medications: Ceftriaxone IV, acetaminophen PRN, methadone 75 mg PO daily

VS: HR 118; BP 129/35 RR: 18; on 2L O2

Relevant labs: UDS positive for cannabis, methadone metabolite; CBC, CMP, LFT’s wnl. EKG with QTc of 457.

Props Needed:

- Luggage
- A couple clothing items or personal belongings
- IV pole with IV

Information for SP:

Initially when you arrived in the hospital, you were very sick and had a fever/chest pain. As today you are feeling better and noticed that it is almost when your rent is due, you started to panic that you are going to miss paying your rent. You need to leave the hospital to check you received a check from the government to pay your rent as you are already behind and are worried you will be evicted. You have tried to express your needs to the hospital staff, and do not feel they understand the severity of the situation with your housing. When you tried to express the situation to the nurse, the nurse was in the middle of documenting and seemed like they were not giving you their full attention (looking at the screen, saying “ok” in the same tone after everything you said…). The nurse responded that the doctors wanted you to stay in the hospital for 5 weeks to complete an antibiotic course, which seems too long to you as you are feeling better. You think it is also unreasonable that you are not allowed to smoke as you are stressed, and smoking cigarettes helps to relieve this.

Case Algorithm (Basic Structure):

| Student respects personal space (example, distance x2, open body language, modulates volume, establish eye contact) | Decrease intensity |
| --- | --- |
| Introduces self, orients patient to role and reassures patient | Decrease intensity |
| Identifies wants/feelings  Example: “how can I help” “what can I do to help you get through this?” | Decrease intensity significantly |
| Offers choices and optimism  Example: treatment of nicotine use disorder, exploring if anyone else can check for the check | Decrease intensity |
| Uses provocative language  Example: “You need to calm down” “I am not going to tell you again.” | Increase intensity |
| Argues with patient | Increase intensity |

*Decrease intensity defined as: decreasing volume, more neutral tone of voice, sitting down rather than standing

*Increase intensity defined as: increasing volume, more irritable tone, big gestures, pacing, standing up. Swearing is okay.

*Please note: Resist leaning forward or over the student/interviewer

Immediately when the student enters the room, do not allow them to introduce themselves. State: “*I’m leaving right now and you can’t stop me.”* [Maintain escalation for 2 questions, student should feel the tension. SP should be pacing, packing up things.]

Student responds that they would like to help you. You state, “*You can’t help me, you are just like the rest of them. In your big white coat, thinking you are better than everyone. I told you already I am leaving now.”*

If the student validates your concerns (ex “things have been very stressful, and I want to make sure you receive the medical care that you deserve.”), start to de-escalate. “*No one here is listening to me. I need to leave to pay my rent or else I am going to lose my house. Do you want me to be homeless?” [tone can be changed to escalate vs de-escalate; example angry vs on verge of tears]*

Student responds with validating statement (ex. “That is really concerning and we certainly do not want you to lose your housing. Can you tell me more about that so that we can problem solve together?”)

You state, “*I lost my job a few months ago and I haven’t been able to pay rent. If I don’t pay this month’s rent they said I could be evicted.*” If the student continues to explore you can elaborate with: *I don’t know if I have enough money. I was supposed to get a check from the government but I’ve been here the last few days. I just can’t be homeless.*”

When discussing recommendation to be in the hospital for 5 weeks, state: *“What if I were a high powered executive or a doctor or lawyer? I couldn’t take 5 weeks off work!”*

Student offers choices/optimism (ex. “I can appreciate the urgency of this situation. Our social worker has expertise in problem solving these types of situations and I think could really help. Would you be open to discussing with them?”) You say, “*Sure, I’ll try anything at this point.”*

*Student asks if patient is willing to stay in the hospital and you say “No. Not completely.”* This is the second hurdle if the student skillfully offers you choices/optimism and de-escalates your concern about the rent. *“I want to go outside and smoke a cigarette. I’m stressed. And they say that I can’t because of this (points to PICC line).” “I have a right to go outside, this is not jail. I’ve seen other patients with this go outside and smoke.”*

Student responds that it is hospital policy that a patient may not go outside with this particular line, especially while the medications are running as there could be potential for adverse effects. Additionally, there is a risk of fire with smoking while on oxygen. You say, “*This is bullshit. I am not in jail and I want to smoke.”*

Student offers choices of nicotine replacement and validates difficulty in withdrawing from nicotine. Offer optimism that while it initially will be uncomfortable, treatment will help with symptoms. You say, *“I came here to get help and I feel like I’m feeling worse in the hospital. I just want to feel better.”*

Student validates and references motivation of patient to get help and get better. You say, “*I do want to get better. Okay I will try what you are recommending.”*

Notes:

*Student may ask if there is anyone at home or a family member/friend to check in on the check. You have limited supports, and don’t want to involve your [insert family member of SP choice and reason for not wanting to involve them] if possible.

*Student may ask if you understand the reason for 4-week duration of hospital course. You feel you are feeling better and no one has updated you on why a 4 week antibiotic inpatient course is needed. You did not know the severity of your condition, and the consequences of not finishing the antibiotic course (risk of re-infection/resistance, sepsis, death).

*You are hungry and have not eaten much the last few days as you were feeling unwell. If the student inquires about this, request a meal, and this helps you to de-escalate/feel your needs are heard.

**Clinical Scenario 2:** [OUTPATIENT] 20 minutes

Observing student reads this prompt to the interviewing student: You are at your clinic evaluating Sam for a general medicine follow up visit for insomnia. You look at the last note approximately 1 month ago and sleep hygiene was recommended as the plan. Specifically, it was recommended to decrease caffeine intake, eliminate daytime naps, and wake up consistently at the same time daily.

Case Overview:

Sam Scully is a 25-55 yo person who presents to your clinic as a follow up. During the course of the evaluation, Sam shares he has been experiencing insomnia over the last 6 months, which has not improved. He is frustrated, as his symptoms have not improved and he has a big presentation upcoming for work that has guaranteed media coverage and could help him in his job promotion if he does well.

PMHx: HTN

Past psychiatric Hx: None

Substance Use Disorder history:

Nicotine: 1 ppd for 30 years

Opioids: none

ETOH: 3-4 beers a week

Crack/cocaine: denies

Cannabis: none

Amphetamines: denies

Hallucinogens: denies

Caffeine: 6-7 cups of coffee a day

Allergies: none

Current medications: Amlodipine, Tylenol PM for sleep

VS: HR 80; BP 135/86 RR: 18. BMI of 29.6

Information for SP:

You are upset as you feel the doctor is not listening to your needs and not taking your symptoms seriously. This is your second visit about insomnia. You are frustrated as the insomnia has been affecting your work, and you work in a high stress job as a lawyer. You feel the doctor is withholding medication that could help you and be a quick fix so that you can function better at work. Further, your physician friend recommended that you be prescribed xanax, so you don’t understand why the doctor is wasting your time with the same history.

Sleep history:

Time in bed: 11 pm

Time falls asleep: 1 am

Time wakes up: 6 am

Daytime naps: after work for 1-2 hours

Nighttime awakenings: 1-2 times

Time to fall back asleep: not sure

What occurs when wake up: need to use restroom, then lays in bed on the phone tossing and turning

Caffeine intake: 6-7 cups a day, last at 5 pm

ETOH intake: weekends, 5-6 beers

Snoring: does not know as patient sleeps alone

Nightmares: denies

Abnormal movements (ex. Restless leg): denies

Pets in bed: dog sleeps in bed with patient

Props:

- SP dressed in suit or business attire
- Cell Phone

Case Algorithm (Basic Structure):

| Student respects personal space (example, distance x2, open body language, modulates volume, establish eye contact) | Decrease intensity |
| --- | --- |
| Introduces self, orients patient to role and reassures patient | Decrease intensity |
| Identifies wants/feelings  Example: “how can I help” “what can I do to help you get through this?” | Decrease intensity significantly |
| Offers choices and optimism  Example: options for evaluation of sleep (ex for sleep apnea) | Decrease intensity |
| Uses provocative language  Example: “You need to calm down” “I am not going to tell you again.” | Increase intensity |
| Argues with patient | Increase intensity |

*Decrease intensity defined as: decreasing volume, more neutral tone of voice, sitting down rather than standing

*Increase intensity defined as: increasing volume, more irritable tone, big gestures, pacing, standing up. Swearing is okay.

*Please note: Resist leaning forward or over the student/interviewer.

Sample Script:

Student introduces self. If student says “Hi Sam,” you say “it’s Samuel.” You respond, “*Doc, I really need something to help me sleep. I am desperate. I have a big presentation coming up for my firm and I can’t be this tired.”*

Student responds asking about aspect of sleep history. You roll your eyes, throw hands in the air, exasperated say “We’ve been over this. I can’t fall asleep, and I am tired all the time. I can’t sleep and I’m exhausted. I need a sleeping pill.”

- If the student states a validating statement instead (ex “It is frustrating to not have sleep.”), you say “*You are damn right it is and I need a pill to help me.”*
- If the student starts to ask about the medication you are interested in or have tried, say, “I don’t like to take pills but this can’t go on. My doctor friend with years of experience also agreed that I need Xanax. I’m like the easiest case for you doc and this is the quickest appointment.” You have not trialed any medications for sleep.

Student tries to explain rationale for sleep history. You interrupt and say (stay frustrated for 2 questions), “Look doc, I don’t have time for this and neither do you. Let’s just order the med and be done with it so we can both get on with our day.” “I don’t see how this is relevant. I just need a pill.” “I tried the sleep hygiene stuff. It didn’t work. Even my physician friend who has been practicing for years thinks that I need a pill called Xanax.”

- If the student asks what exactly you tried for sleep hygiene, you say “It says it all in the chart. Did you read it?”
- If student makes a validating statement, then state, “I tried the not napping but I am so exhausted and I can’t do it. I need the caffeine to help me stay awake and I can’t cut back.” “These are good things in theory but they are not practical. You don’t understand the pressure I am under at work.”

Student makes a validating statement and explains rationale for sleep history (ex. The sleep history is really important so that we can make sure there are not other factors and illnesses contributing to poor sleep like sleep apnea. Plus, our medications help with falling asleep, but less with staying asleep and depending on what you are struggling with, I want to make sure we have a good plan in place that will lead to success.) You say, “Ok that makes sense, no one explained it like that before.”

- If the student does not explain the rationale fully (ex. We need a good history so that we know what medication to prescribe), then at the end, push for the medication again (“I did what you said, and answered your questions. Now it is your turn to keep your promise about the medication.”)

Student takes sleep history as per above.

Notes:

*SP cannot ask for MD or someone with more experience to enter the room/take over.

Note to SP: We want the student to feel uncomfortable by dropping hints that question their authority but not to the point that you request the attending or MD to evaluate you.

**REFERENCES:**

Vestal HS, Sowden G, Nejad S, et al. Simulation-based training for residents in the management of acute agitation: a cluster randomized controlled trial. *Acad Psychiatry*. 2017;41(1):62-67. doi: 10.1007/s40596-016-0559-2.

<https://static-content.springer.com/esm/art%3A10.1007%2Fs40596-016-0559-2/MediaObjects/40596_2016_559_MOESM1_ESM.pdf>

Richmond JS, Berlin JS, Fishkind AB, et al. Verbal de-escalation of the agitated patient: consensus statement of the american association for Emergency Psychiatry Project BETA De-Escalation Workgroup. *West J Emerg Med*. 2012;13(1):17–25. <https://doi.org/10.5811/westjem.2011.9.6864>
