# Supplementary material for: A Verbal De-escalation Standardized Patient Workshop for Third- and Fourth-Year Medical Students
Source: MedEdPORTAL. 2024 Jul 19;20:11417. doi: 10.15766/mep_2374-8265.11417 (PMC11258212; doi:10.15766/mep_2374-8265.11417)
Supplement: Supplementary file 1 — SP Cases.docxLogistics.docxWorkshop.docxVerbal De-escalation Primer.pptxCase 1 Prompt.docxCase 2 Prompt.docxSP Learner Feedback.docxInstructions for Observing Learner-Led Debrief.docxStudent Handout.docxStudent Evaluation Form.docx [file mep_2374-8265.11417-s001.zip › D. Verbal De-escalation Primer.pptx]

## Slide 1
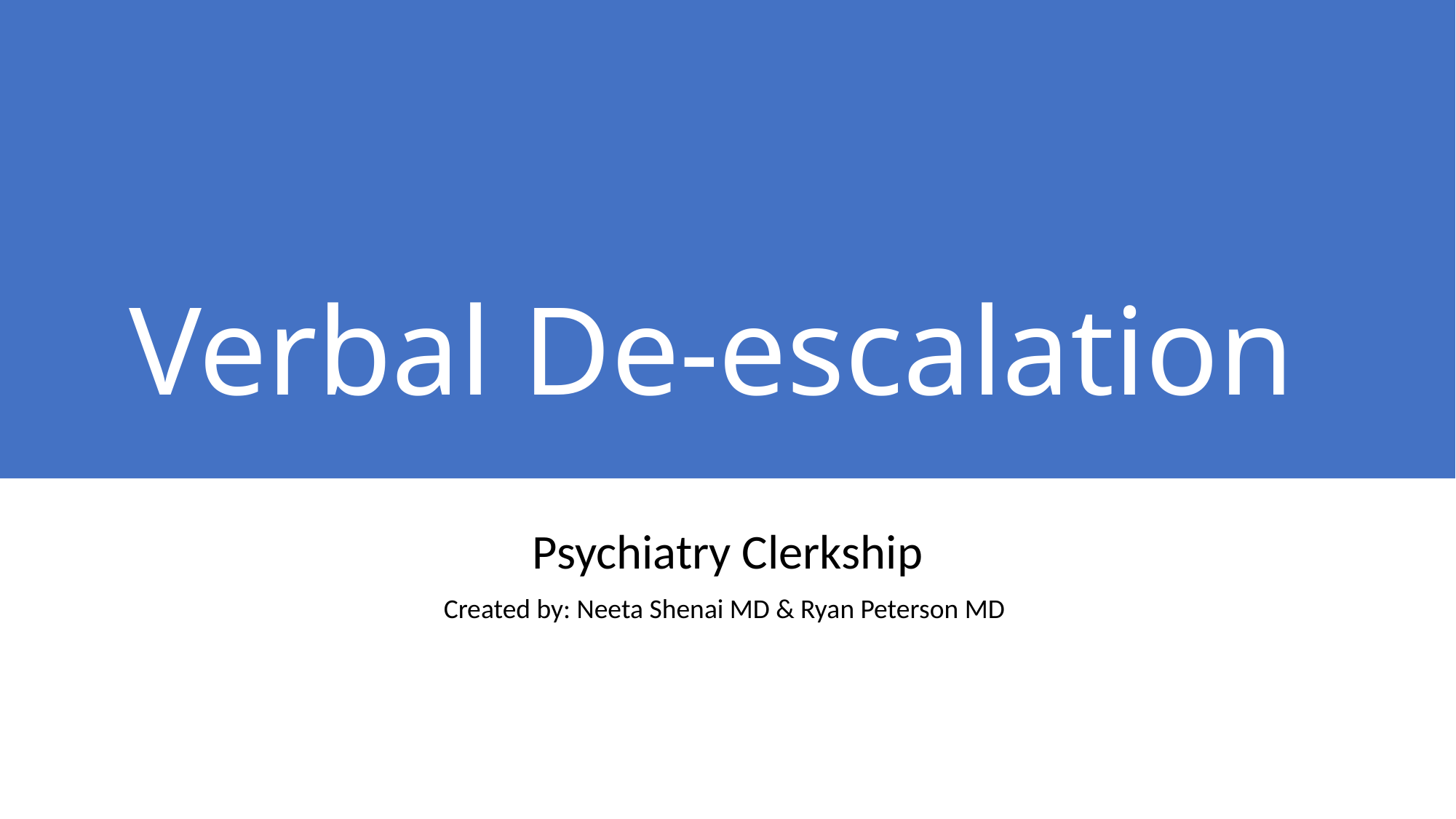

# Verbal De-escalation
Psychiatry Clerkship
Created by: Neeta Shenai MD & Ryan Peterson MD

## Slide 2
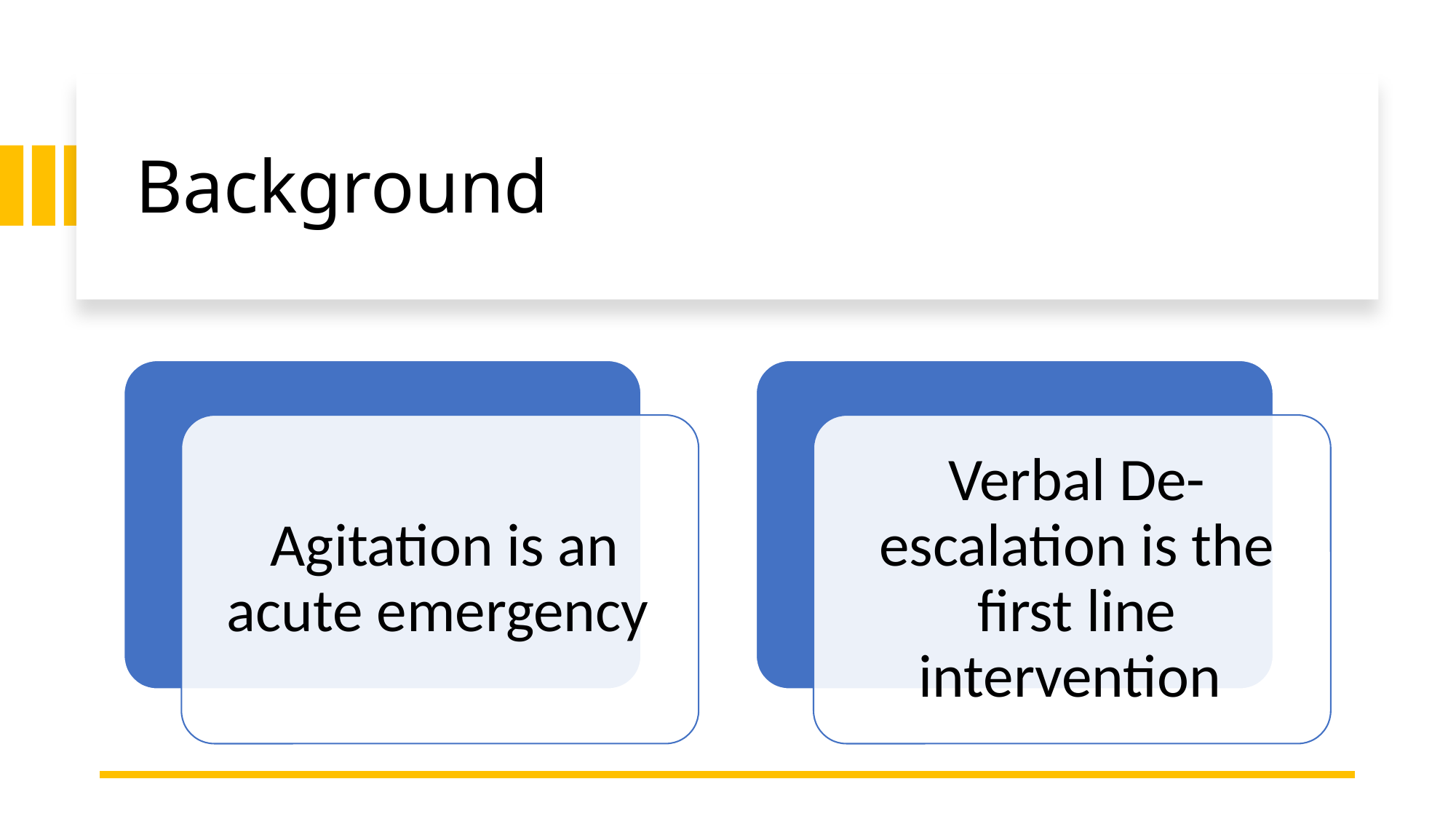

# Background

## Slide 3
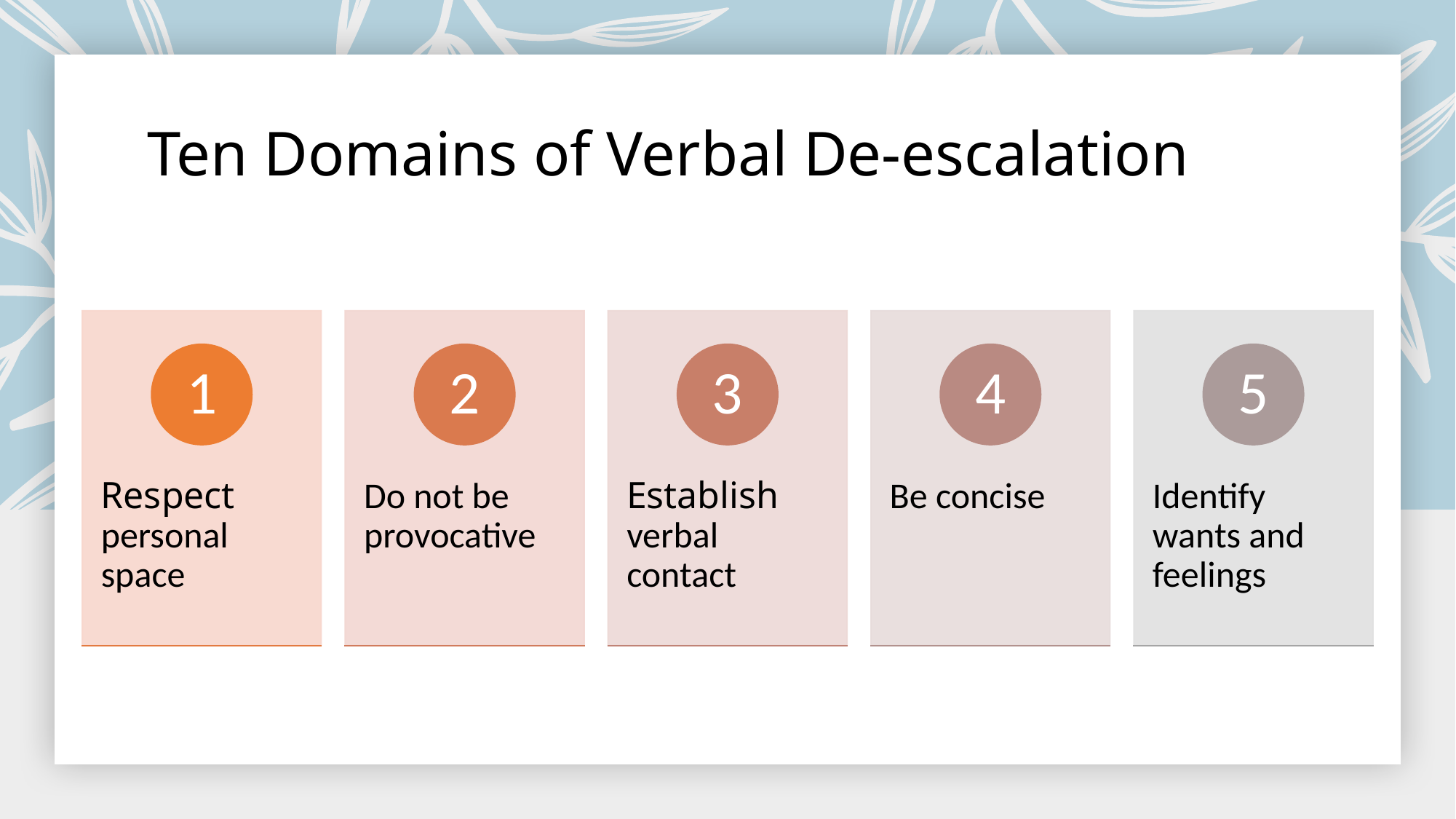

# Ten Domains of Verbal De-escalation

## Slide 4
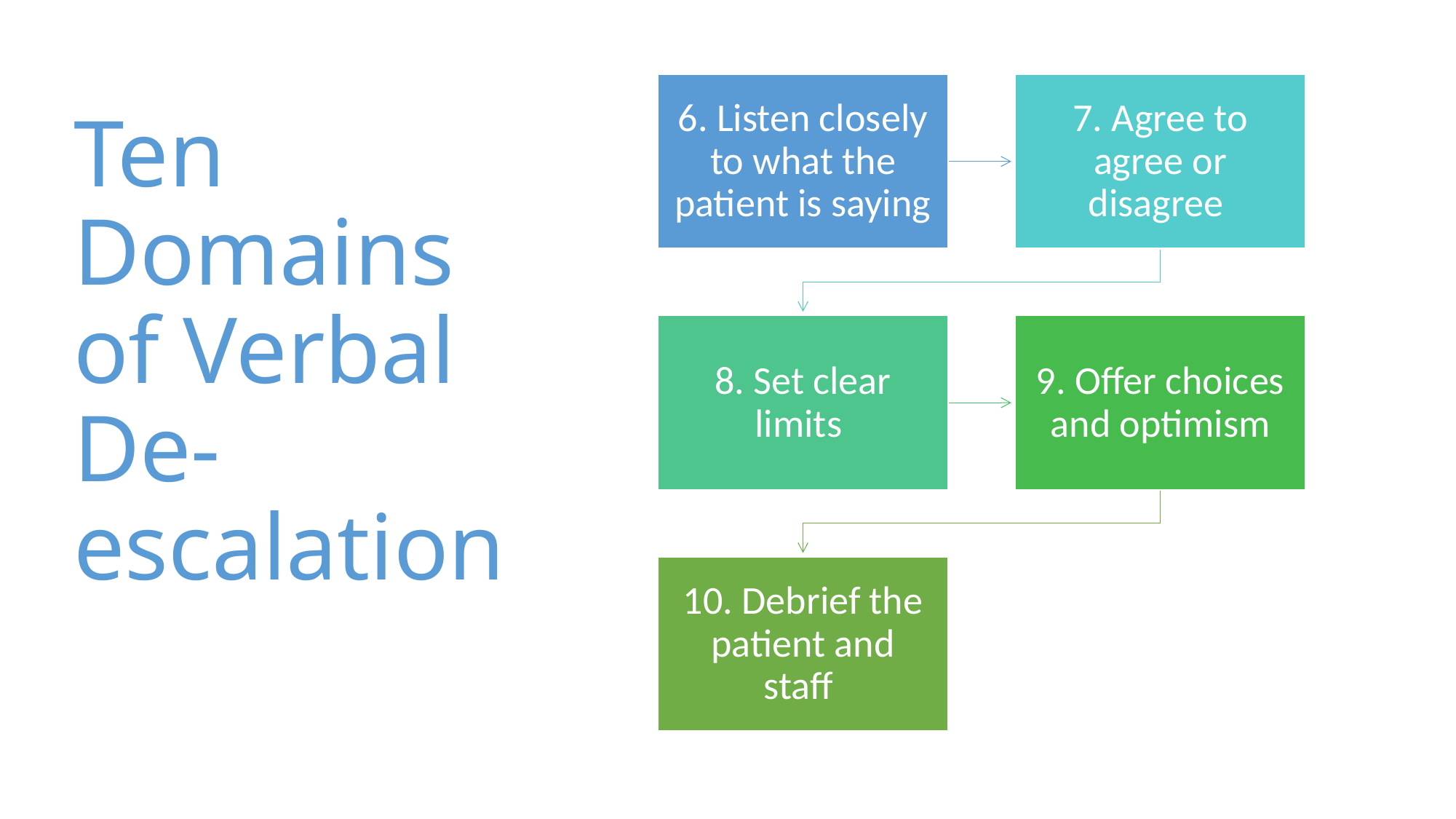

# Ten Domains of Verbal De-escalation

## Slide 5
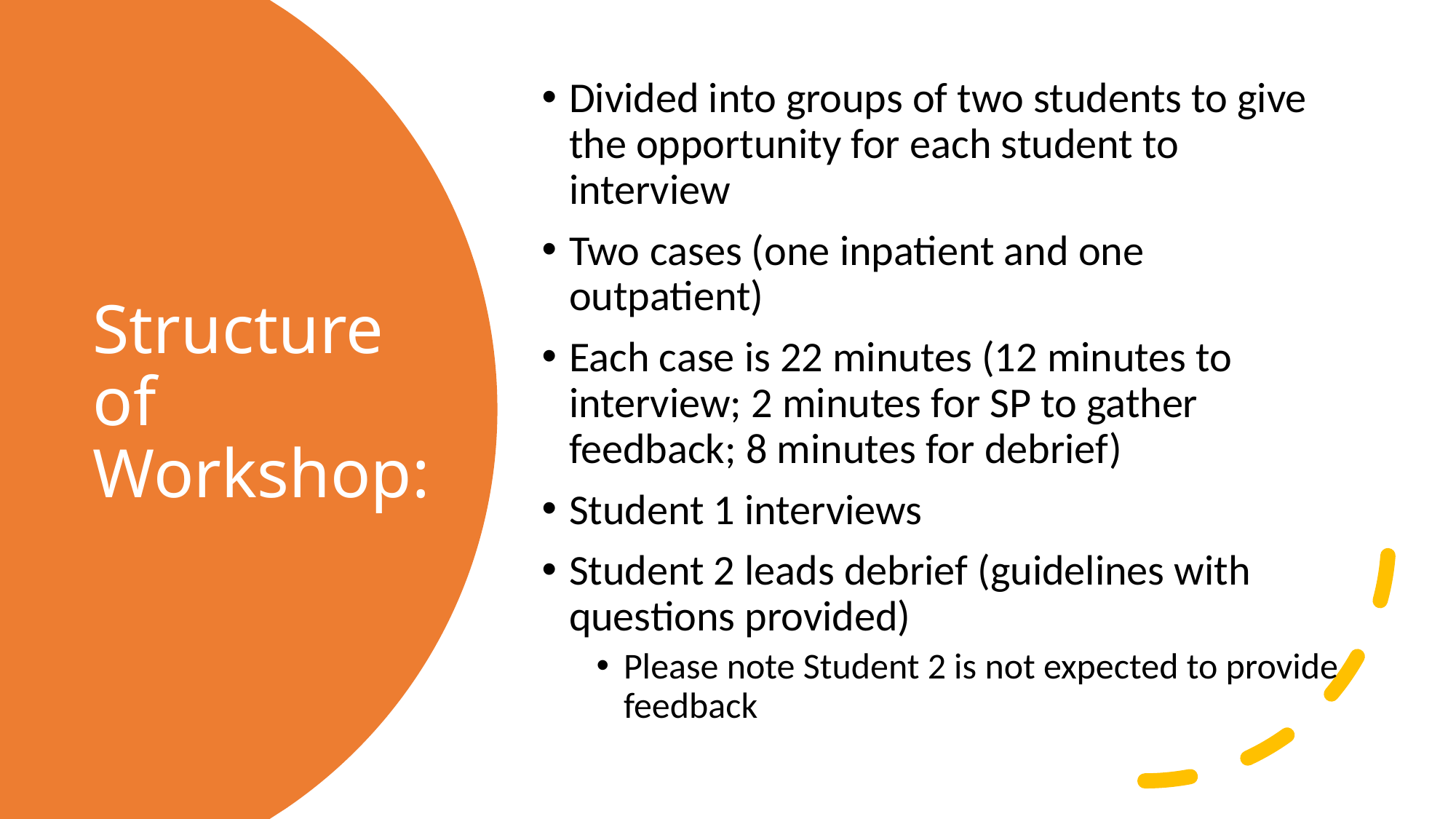

Divided into groups of two students to give the opportunity for each student to interview
Two cases (one inpatient and one outpatient)
Each case is 22 minutes (12 minutes to interview; 2 minutes for SP to gather feedback; 8 minutes for debrief)
Student 1 interviews
Student 2 leads debrief (guidelines with questions provided)
Please note Student 2 is not expected to provide feedback
# Structure of Workshop:

## Slide 6
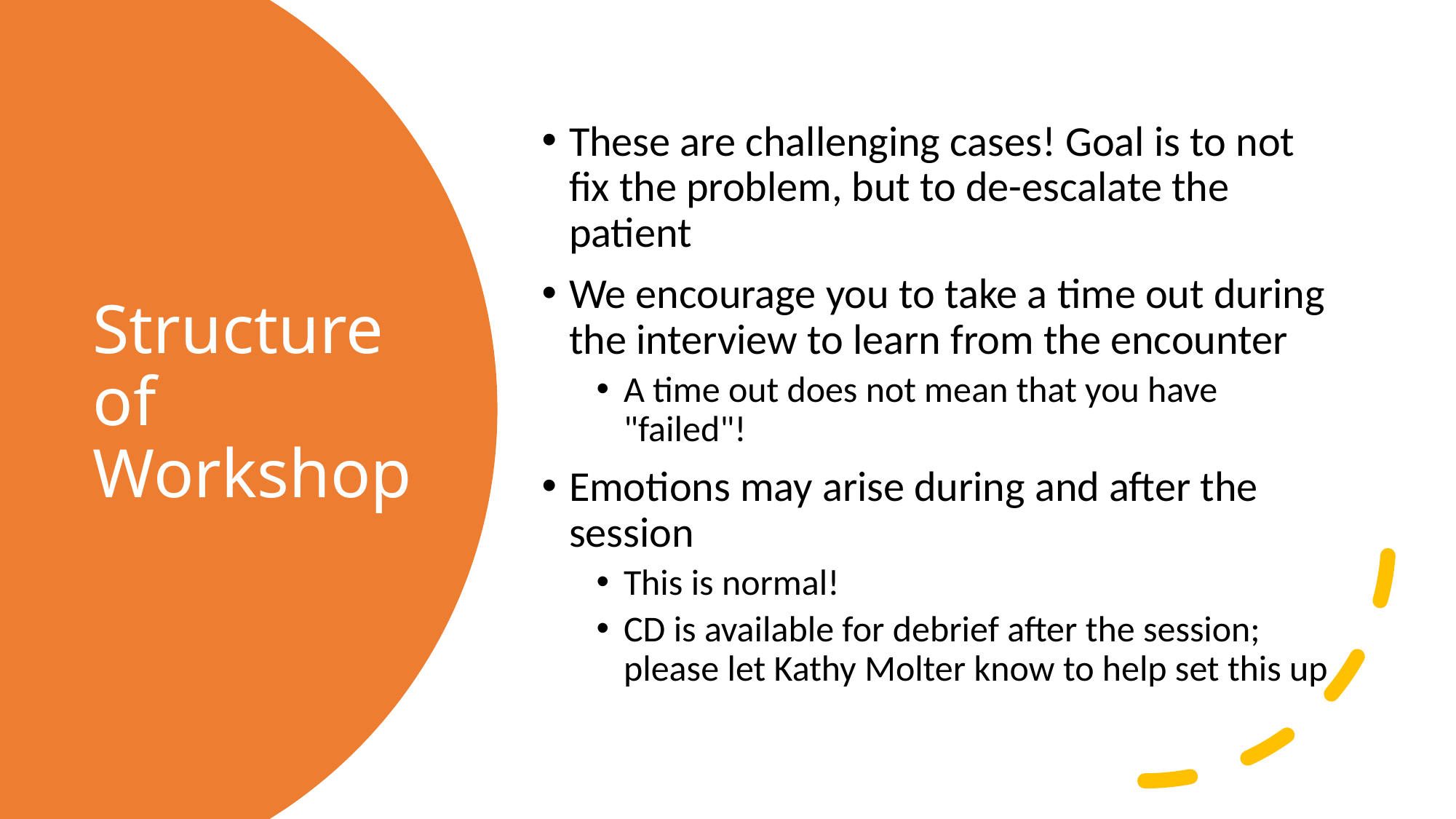

These are challenging cases! Goal is to not fix the problem, but to de-escalate the patient
We encourage you to take a time out during the interview to learn from the encounter
A time out does not mean that you have "failed"!
Emotions may arise during and after the session
This is normal!
CD is available for debrief after the session; please let Kathy Molter know to help set this up
# Structure of Workshop
