# Supplementary material for: A Verbal De-escalation Standardized Patient Workshop for Third- and Fourth-Year Medical Students
Source: MedEdPORTAL. 2024 Jul 19;20:11417. doi: 10.15766/mep_2374-8265.11417 (PMC11258212; doi:10.15766/mep_2374-8265.11417)
Supplement: Supplementary file 1 — SP Cases.docxLogistics.docxWorkshop.docxVerbal De-escalation Primer.pptxCase 1 Prompt.docxCase 2 Prompt.docxSP Learner Feedback.docxInstructions for Observing Learner-Led Debrief.docxStudent Handout.docxStudent Evaluation Form.docx [file mep_2374-8265.11417-s001.zip › G. SP Learner Feedback.docx]

Appendix G

SP’s Real Name: ______________________ Student Name: ______________________

Date: _________________________

What went well about the interview? What could have improved?”

- You respected my personal space. (Kept the bed between us while we talked)
- You were not provocative physically or verbally.

a. demeanor and voice were calm,

b. hands visible and unclenched,

c. eye contact was direct but not staring or aggressive,

d. body language was open and non-confrontational,

e. you did not insult or patronize me or my concerns

- You verbally connected with me when you;
  a. asked me how I’d like to be addressed,
  b. clarified your role,
  c. explained that you are here to listen to my concerns, “I may not be able to fix everything, but I can listen and try to help”

d. Validated the seriousness of my concerns/situation. (Concern about getting kicked out of housing)

- When you used active listening I felt heard and respected:
  a. listened closely (physical cues and non- verbal utterances)
  b. repeated my words back to me
  c. validated my concerns
  d. gave me a chance to clarify or correct stated or implied emotions …“Tell me if I have this right”
- You identified my wants, expectations, and feelings. (You validated my concerns about housing/rent and the need for help in taking care of it)
- When you explained concisely using short sentences and simple vocabulary, it gave me time to process and understand the rationale behind needing more info about why you are upset.

“If I don’t know the details, it’ll be hard to get you the help you need.”

- You explored in detail my housing/ financial concerns and my desire to leave the floor to smoke.
- Agree or agree to disagree.
  a. Find something about the situation/ patients position with which you CAN agree (see article)

b. if there is no way to honestly agree with the patient, respectfully agree to disagree

- Set clear limits if the patient’s behavior is causing the clinician to be frightened or disrespected.
- Offer choices and optimism- Offer realistic options or choices, including acts of kindness (food, water, phone, …) You addressed both rent payment ideas (speak with social worker?) and nicotine withdrawal comfort (nicotine replacement options, speak with tobacco treatment team).

**Instructions for Feedback Case 2 (Outpatient)**

SP’s Real Name: ______________________ Student Name: ______________________

Date: _________________________

What went well about the interview? What could have improved?

- You respected my personal space. (Kept the table between us while we talked)
- You were not provocative physically or verbally.

a. demeanor and voice were calm,

b. hands visible and unclenched,

c. eye contact was direct but not staring or aggressive,

d. body language was open and non-confrontational,

e. you did not insult or patronize me or my concerns

- You verbally connected with me when you;
  a. asked me how I’d like to be addressed,
  b. clarified your role,
  c. explained that you are here to listen to my concerns, “I may not be able to fix everything, but I can listen and try to help”

d. Validated the seriousness of my concerns/situation.

- When you used active listening I felt heard and respected:
  a. listened closely (physical cues and non- verbal utterances)
  b. repeated my words back to me
  c. validated my concerns
  d. gave me a chance to clarify or correct stated or implied emotions …“Tell me if I have this right”
- You identified my wants, expectations, and feelings. (Frustration with “sleep hygiene”, impact of sleep on work/stress, desire to have medication)
- When you explained concisely using short sentences and simple vocabulary, it gave me time to process and understand the rationale behind needing more info about my sleep history.

“If I don’t know the details, it’ll be hard to get you the help you need.”

- You explored in detail my sleep history (obtained at least 3 of the following)
  - Time in bed: 11 pm
  - Time falls asleep: 1 am
  - Time wakes up: 6 am
  - Daytime naps: after work for 1-2 hours
  - Nighttime awakenings: 1-2 times
  - Time to fall back asleep: not sure
  - What occurs when wake up: need to use restroom, then lays in bed on the phone tossing and turning
  - Caffeine intake: 6-7 cups a day, last at 5 pm
  - ETOH intake: weekends, 5-6 beers
  - Snoring: does not know as patient sleeps alone
  - Pets in bed: dog sleeps in bed with patient
- You explored what sleep hygiene techniques I tried.
- Agree or agree to disagree.
  a. Find something about the situation/ patients position with which you CAN agree (see article)

b. if there is no way to honestly agree with the patient, respectfully agree to disagree

- Set clear limits if the patient’s behavior is causing the clinician to be frightened or disrespected. This includes not offering alprazolam as this is not clinically appropriate.
- Offer choices and optimism- Offer realistic options or choices, including acts of kindness (food, water, phone, …)
