# Supplementary material for: A Verbal De-escalation Standardized Patient Workshop for Third- and Fourth-Year Medical Students
Source: MedEdPORTAL. 2024 Jul 19;20:11417. doi: 10.15766/mep_2374-8265.11417 (PMC11258212; doi:10.15766/mep_2374-8265.11417)
Supplement: Supplementary file 1 — SP Cases.docxLogistics.docxWorkshop.docxVerbal De-escalation Primer.pptxCase 1 Prompt.docxCase 2 Prompt.docxSP Learner Feedback.docxInstructions for Observing Learner-Led Debrief.docxStudent Handout.docxStudent Evaluation Form.docx [file mep_2374-8265.11417-s001.zip › H. Instructions for Observing Learner-Led Debrief.docx]

**Appendix H. Instructions for Observing Learner Lead Debrief**

Student observer is in charge of beginning the feedback:

Should the learner time out:

1) Observer asks Interviewer, “What caused you to time out?”

2) Observer states “Lets return to that in a moment” and asks “what went well during the interview so far?”

3) Observer shares what they saw going well.

4) Observer asks Interviewer, “What do you want to achieve when you go back into the interview?”

5) Observer asks Interviewer, “What skill will you use to achieve that goal?”

6) Observer asks Interviewer, “What exact words will you use when you start again?”

7) Observer instructs “we’ll start again, right before we stopped last time, and you’ll begin with that line. Time in.”

When the interview portion is over due to time:

1) Observer asks Interviewer "What went well during that interview?"

2) Observer asks Patient, "What do you think went well during the interview?"

3) Observer offers what they saw the Interviewer do skillfully.

4) Observer asks Interviewer, "If you could do it over again, what would you do differently?"

5) Observer asks Patient, "What could have improved the interview for you?" or “What did you find challenging about the interview?”

6) Interviewer may ask the Patient (i.e., "When I did ____, how did that make you feel?") or Observer a question (“When I did _____, how did that work?”
