# Supplementary material for: A Verbal De-escalation Standardized Patient Workshop for Third- and Fourth-Year Medical Students
Source: MedEdPORTAL. 2024 Jul 19;20:11417. doi: 10.15766/mep_2374-8265.11417 (PMC11258212; doi:10.15766/mep_2374-8265.11417)
Supplement: Supplementary file 1 — SP Cases.docxLogistics.docxWorkshop.docxVerbal De-escalation Primer.pptxCase 1 Prompt.docxCase 2 Prompt.docxSP Learner Feedback.docxInstructions for Observing Learner-Led Debrief.docxStudent Handout.docxStudent Evaluation Form.docx [file mep_2374-8265.11417-s001.zip › I. Student Handout.docx]

**Appendix I. Verbal De-Escalation Workshop Student Handout**

Verbal De-Escalation Techniques

1. Respect personal space
   1. Anger = Distance x2
   2. No excessive eye contact, open body language, guide volume, pace with patient, take breaks
2. Do not be provocative
   1. Phrases to avoid: “I am not going to tell you again”; “You need to calm down”
3. Establish verbal contact
   1. Only one person interacts, introduce yourself, orient and reassure
4. Be concise
5. Identify wants/feelings
   1. “How can I help”; “What can we do to help get you through this?”
6. Listen closely to what the patient is saying
7. Agree to agree or disagree
   1. State your decision clearly/succinctly; don’t argue
8. Set clear limits without escalating
9. Offer choices and optimism
10. Debrief the patient and with staff
    1. Provide support and gain knowledge
