# Supplementary material for: A Verbal De-escalation Standardized Patient Workshop for Third- and Fourth-Year Medical Students
Source: MedEdPORTAL. 2024 Jul 19;20:11417. doi: 10.15766/mep_2374-8265.11417 (PMC11258212; doi:10.15766/mep_2374-8265.11417)
Supplement: Supplementary file 1 — SP Cases.docxLogistics.docxWorkshop.docxVerbal De-escalation Primer.pptxCase 1 Prompt.docxCase 2 Prompt.docxSP Learner Feedback.docxInstructions for Observing Learner-Led Debrief.docxStudent Handout.docxStudent Evaluation Form.docx [file mep_2374-8265.11417-s001.zip › J. Student Evaluation Form.docx]

Appendix J: Verbal De-escalation Student Evaluation Form

1. The verbal de-escalation workshop met the stated learning objectives.
   1. Strongly agree
   2. Agree
   3. Neutral
   4. Disagree
   5. Strongly disagree
2. What would you rate the educational quality of the De-Escalation Experience?
   1. A (best)
   2. B
   3. C
   4. D (worst)
3. What was the best part of the verbal de-escalation workshop? (free text)
4. What would you recommend to make the verbal de-escalation workshop better? (free text)
