# Supplementary material for: A Verbal De-escalation Standardized Patient Workshop for Third- and Fourth-Year Medical Students
Source: MedEdPORTAL. 2024 Jul 19;20:11417. doi: 10.15766/mep_2374-8265.11417 (PMC11258212; doi:10.15766/mep_2374-8265.11417)
Supplement: Supplementary file 1 — SP Cases.docxLogistics.docxWorkshop.docxVerbal De-escalation Primer.pptxCase 1 Prompt.docxCase 2 Prompt.docxSP Learner Feedback.docxInstructions for Observing Learner-Led Debrief.docxStudent Handout.docxStudent Evaluation Form.docx [file mep_2374-8265.11417-s001.zip › A. SP Cases.docx]

**Appendix A: SP Cases**

**Inpatient Case**

Date: May 1^st^, 2022

Primary Case Author: Neeta Shenai, MD

Secondary Case Author: Catherine Gowl, Val Fulmer, Reed Van Deusen MD

Standardized Patient Educator: Val Fulmer, Catherine Gowl

Name of Case: “Inpatient Case”

Name of Educational and/or Assessment Activity: Verbal De-escalation Standardized Patient Workshop

Patient Name: Taylor Jackson

Chief Complaint: “I want to leave.”

Most Likely Diagnosis and Differential With Rationale From History and/or Physical Exam: Infectious endocarditis

Challenge Question: none

Domains: Check all that apply

1. Professionalism X
2. Communication and Interpersonal Skills X
3. Medical History
4. Physical Exam
5. Shared Decision-Making X
6. Patient Education X
7. Clinical Reasoning
8. Documentation
9. Handoff
10. Presentation

Other:

Type and Level of Learner: medical student (MS3, MS4’s)

Case Objectives: Please list specific objectives for each of the domains you have checked above:

1. Apply the techniques of verbal de-escalation to a realistic, simulated scenario with a standardized patient displaying anger and agitation.

2. Identify one element of the encounter that went well and one element that can be improved upon through a student-led reflection debrief.

| SETTING: outpatient, in patient, ED, home, nursing home, rehab, group, etc. | Inpatient |
| --- | --- |
| PATIENT PROFILE: Information about the “patient” that helps select an SP and helps the learner get an understanding of them as a person. SP will know more information about the patient than learner will ever ask but allows SP to portray a fully developed patient personality. If none of the items below are particulars for the case, please write “all may be used.” | |
| Age range | 20-55 years old |
| Religious/spiritual background | Any |
| Sex (e.g., male, female, intersex, transwoman, transman) | Any |
| Sexual orientation (e.g., heterosexual, lesbian, gay, bisexual, pansexual, queer, asexual) | Any |
| Gender expression (e.g., man, woman, genderqueer) | Any |
| Race and ethnicity | Any |
| Physical description (e.g., BMI, height range) | None |
| Physical limitations | None |
| Patient appearance (e.g., disheveled, hospital gown, business casual, casual) | Hospital gown |
| Moulage + location (e.g., none, bruises, scars, body piercing, tattoos) | None |
| Affect (e.g., pleasant, cooperative) | Upset, irritable |
| Family group (e.g., who is family, who they live with) | Limited support |
| Education | Any |
| Level of health literacy | Any |
| Employment, if any - present and past, noting any current stresses | Any |
| Home/homeless - type of dwelling, number of stories, owned or rented | Renting and need to pay your rent, otherwise you will be evicted. |
| Financial situation - any current stresses | Financial insecurity present |
| Insurance status (e.g., un/under/insured, public/private, HMO/PPO) | Insured |
| Habits (i.e., diet, exercise, caffeine, smoking, alcohol, drugs) | Smoking daily, cannabis “here and there”, history of opioid use disorder |
| Activities (i.e., hobbies, sports, clubs, friends) | Any |
| Typical day - what is the usual daily routine | Any |

| CASE INFORMATION | |
| --- | --- |
| Chief Concern: What the patient will say when greeted by the student. The patient’s primary reason for seeking medical care often stated in their own words. | “*I’m leaving right now and you can’t stop me.”* |
| Additional Concerns: Other, if any, concerns the patient has today (i.e., symptoms, requests, expectations, etc.) that will become part of set agenda. | “*You can’t help me, you are just like the rest of them. In your big white coat, thinking you are better than everyone. I told you already I am leaving now.”* |
| THE PATIENT’S STORY: The SP will be asked to tell their symptom story and the personal and emotion impact for each of their concerns. You will want to write this in the patient’s voice. The symptom story should be able to answer this question: “Tell me more about [chief concern/additional concern], starting at the beginning and bringing me up to now.”    The personal context should be able to answer questions concerning the broader personal/psychosocial context of symptoms, especially the patient’s beliefs/attributions.    The emotional context should be able to ask how are you doing with this, how does this make you feel, how has this affected you emotionally? IMPACT: How has this affected your life? How has this been for your family? | Initially when you arrived in the hospital, you were very sick and had a fever/chest pain. As today you are feeling better, and noticed that it is almost when your rent is due, you started to panic that you are going to miss paying your rent. You need to leave the hospital to check you received a check from the government to pay your rent as you are already behind and are worried you will be evicted. You have tried to express your needs to the hospital staff, and do not feel they understand the severity of the situation with your housing. When you tried to express the situation to the nurse, the nurse was in the middle of documenting and seemed like they were not giving you their full attention (looking at the screen, saying “ok” in the same tone after everything you said…). The nurse responded that the doctors wanted you to stay in the hospital for 5 weeks to complete an antibiotic course, which seems too long to you as you are feeling better. You think it is also unreasonable that you are not allowed to smoke as you are stressed, and smoking cigarettes helps to relieve this. |
| HISTORY OF PRESENT ILLNESS: Although some of the HPI will be given in the patient’s symptom story, the learners will expand the story during the direct question section. Below, describe the detailed history, usually about the chief concern, which the student must develop in order to make a useful assessment of the problem: | |
| Onset (when; gradual or sudden) | sudden |
| Setting (what was going on or where was patient when symptoms first noticed?) | At apartment |
| Duration (how long) | Few days |
| Time relationships (frequency, constant or intermittent) | Constant initially, better now |
| Location | chest |
| Radiation | none |
| Quality | sharp |
| Amount | N/A |
| Aggravated by what | N/A |
| Relieved by what | N/A |
| Associated with what | N/A |
| Attitude (what does the patient think is the problem, and how do they feel about it) | Thinks it is improving |
| Overall course | Improving |
| REVIEW OF SYSTEMS: Significant positives and negatives | |
|  | Shortness of breath improving |
|  |  |
|  |  |
|  |  |
|  |  |
| Past medical history |  |
| Medication allergies (name and reaction) | None |
| Environmental allergies (name and reaction) | None |
| Illnesses | None |
| Vaccinations | N/A |
| Surgeries | None |
| Accidents/injuries/trauma | None |
| Hospitalization | N/A |
|  | |
| Inclusive sexual and reproductive history | |
| Sexual practices  Sexual partners  Protection: Use of safer sex practices  Use of birth control if appropriate  Risk of intimate partner violence | N/A |
| OB/GYN history | Age of onset of menses N/A  Age of menopause N/A  Number of pregnancies N/A  Number of live births N/A  Number of miscarriages N/A  Number of abortions N/A |
| Medications | Ceftriaxone IV, acetaminophen PRN, methadone 75 mg PO daily |
| Immunizations | 1. All up to date |
| Tobacco products:   1. Cigarettes 2. Cigar 3. Pipe 4. Chew 5. E-cigarettes | Current daily smoker 1 ppd for 20 years |
| Alcohol   1. Beer 2. Wine 3. Liquor 4. Other | None currently |
| Drugs   1. Weed 2. Cocaine 3. Heroin 4. Meth 5. IV 6. Inhalants 7. Other | Cannabis use “here and there”  Opioids: history of daily use and has been stable for a few years on methadone |
| Diet (describe) | N/A |
| Exercise (describe) | N/A |
| List any other important social history or information important to this case | About to be evicted if patient does not pay rent |
| Family history |  |
| Mother, father, siblings, grandparents, and other significant findings | N/A |
|  |  |
| Physical Exam - List exam maneuvers expected for this case and any abnormal findings that SP will simulate. (tenderness, hyper-hypo reflex, rebound, weakness, etc.):  None | |
| PHYSICAL EXAM FINDINGS |  |
| 1. Written in layperson’s terms | NA |
| 1. General appearance - affect, appearance, position of patient at opening (i.e., sitting, lying down, holding abdomen, etc.) | Standing up, angry affect |
| 1. Vital signs | HR 118; BP 129/35 RR: 18; on 2L O2 |
| 1. Specific findings and affect | Angry affect |
| 1. Response to certain physical movements | None |
|  |  |
| DIAGNOSIS AND DIFFERENTIAL |  |
| Diagnosis with support from positive and negative history and PE findings | Infectious endocarditis |
| Differential with support from positive and negative history and PE findings |  |
|  |  |
| MANAGEMENT OR DIAGNOSTIC PLAN | None as the purpose is to de-escalate the patient. The student can offer choices such as meeting with a social worker, discussing with staff hospital policy regarding going outside to smoke cigarettes. |
|  |  |
| PROFESSIONALISM ISSUES OR CHALLENGES | The challenge of this case is to verbally de-escalate a patient who is angry and irritable. |

**Outpatient Case**

Date: May 1^st^, 2022

Primary Case Author: Neeta Shenai, MD

Secondary Case Author: Catherine Gowl, Val Fulmer, Reed Van Deusen MD

Standardized Patient Educator: Val Fulmer, Catherine Gowl

Name of Case: “Outpatient Case”

Name of Educational and/or Assessment Activity: Verbal De-escalation Standardized Patient Workshop

Patient Name: Taylor Jackson

Chief Complaint: “I want to leave.”

Most Likely Diagnosis and Differential With Rationale From History and/or Physical Exam: Infectious endocarditis

Challenge Question: none

Domains: Check all that apply

1. Professionalism X
2. Communication and Interpersonal Skills X
3. Medical History
4. Physical Exam
5. Shared Decision-Making X
6. Patient Education X
7. Clinical Reasoning
8. Documentation
9. Handoff
10. Presentation

Other:

Type and Level of Learner: Medical student (MS3, MS4)

Case Objectives: Please list specific objectives for each of the domains you have checked above:

1. Apply the techniques of verbal de-escalation to a realistic, simulated scenario with a standardized patient displaying anger and agitation.

2. Identify one element of the encounter that went well and one element that can be improved upon through a student-led reflection debrief.

| SETTING: outpatient, in patient, ED, home, nursing home, rehab, group, etc. | Outpatient |
| --- | --- |
| PATIENT PROFILE: Information about the “patient” that helps select an SP and helps the learner get an understanding of them as a person. SP will know more information about the patient than learner will ever ask but allows SP to portray a fully developed patient personality. If none of the items below are particulars for the case, please write “all may be used.” | |
| Age range | 20-55 year old |
| Religious/spiritual background | Any |
| Sex (e.g., male, female, intersex, transwoman, transman) | Any |
| Sexual orientation (e.g., heterosexual, lesbian, gay, bisexual, pansexual, queer, asexual) | Any |
| Gender expression (e.g., man, woman, genderqueer) | Any |
| Race and ethnicity | Any |
| Physical description (e.g., BMI, height range) | Any |
| Physical limitations | None |
| Patient appearance (e.g., disheveled, hospital gown, business casual, casual) | Well dressed, business professional |
| Moulage + location (e.g., none, bruises, scars, body piercing, tattoos) | None |
| Affect (e.g., pleasant, cooperative) | Irritable |
| Family group (e.g., who is family, who they live with) | None |
| Education | Law school |
| Level of health literacy | High |
| Employment, if any - present and past, noting any current stresses | Lawyer, big court case coming up |
| Home/homeless - type of dwelling, number of stories, owned or rented | Home |
| Financial situation - any current stresses | No stressors |
| Insurance status (e.g., un/under/insured, public/private, HMO/PPO) | Insured |
| Habits (i.e., diet, exercise, caffeine, smoking, alcohol, drugs) | High caffeine intake |
| Activities (i.e., hobbies, sports, clubs, friends) | Any |
| Typical day - what is the usual daily routine | At work for most of the day |

| CASE INFORMATION | |
| --- | --- |
| Chief Concern: What the patient will say when greeted by the student. The patient’s primary reason for seeking medical care often stated in their own words. | “it’s Samuel.” “*Doc, I really need something to help me sleep. I am desperate. I have a big presentation coming up for my firm and I can’t be this tired.”* |
| Additional Concerns: Other, if any, concerns the patient has today (i.e., symptoms, requests, expectations, etc.) that will become part of set agenda. | “We’ve been over this. I can’t fall asleep and I am tired all the time. I can’t sleep and I’m exhausted. I need a sleeping pill.” |
| THE PATIENT’S STORY: The SP will be asked to tell their symptom story and the personal and emotion impact for each of their concerns. You will want to write this in the patient’s voice. The symptom story should be able to answer this question: “Tell me more about [chief concern/additional concern], starting at the beginning and bringing me up to now.”    The personal context should be able to answer questions concerning the broader personal/psychosocial context of symptoms, especially the patient’s beliefs/attributions.    The emotional context should be able to ask how are you doing with this, how does this make you feel, how has this affected you emotionally? IMPACT: How has this affected your life? How has this been for your family? | You are upset as you feel the doctor is not listening to your needs and not taking your symptoms seriously. This is your second visit about insomnia. You are frustrated as the insomnia has been affecting your work, and you work in a high stress job as a lawyer. You feel the doctor is withholding medication that could help you and be a quick fix so that you can function better at work. Further, your physician friend recommended that you be prescribed xanax, so you don’t understand why the doctor is wasting your time with the same history. |
| HISTORY OF PRESENT ILLNESS: Although some of the HPI will be given in the patient’s symptom story, the learners will expand the story during the direct question section. Below, describe the detailed history, usually about the chief concern, which the student must develop in order to make a useful assessment of the problem: | |
| Onset (when; gradual or sudden) | Chronic |
| Setting (what was going on or where was patient when symptoms first noticed?) | Home |
| Duration (how long) | Many months |
| Time relationships (frequency, constant or intermittent) | N/A |
| Location | N/A |
| Radiation | N/A |
| Quality | N/A |
| Amount | N/A |
| Aggravated by what | N/A |
| Relieved by what | N/A |
| Associated with what | N/A |
| Attitude (what does the patient think is the problem, and how do they feel about it) | Patient feels that xanax will help their sleep. |
| Overall course | Constant |
| REVIEW OF SYSTEMS: Significant positives and negatives | |
|  | None |
|  |  |
|  |  |
|  |  |
|  |  |
| Past medical history |  |
| Medication allergies (name and reaction) | None |
| Environmental allergies (name and reaction) | None |
| Illnesses | HTN |
| Vaccinations | Up to date |
| Surgeries | None |
| Accidents/injuries/trauma | None |
| Hospitalization | None |
|  | |
| Inclusive sexual and reproductive history | |
| Sexual practices  Sexual partners  Protection: Use of safer sex practices  Use of birth control if appropriate  Risk of intimate partner violence | N/A |
| OB/GYN history | N/A |
| Medications | Amlodipine, tylenol PM for sleep |
| Immunizations | Up to date |
| Tobacco products:   1. Cigarettes 2. Cigar 3. Pipe 4. Chew 5. E-cigarettes | 1 ppd for 30 years |
| Alcohol   1. Beer 2. Wine 3. Liquor 4. Other | 3-4 beers a week |
| Drugs   1. Weed 2. Cocaine 3. Heroin 4. Meth 5. IV 6. Inhalants 7. Other | None |
| Diet (describe) | “eat when I can” |
| Exercise (describe) | None |
| List any other important social history or information important to this case | High stress job |
| Family history |  |
| Mother, father, siblings, grandparents, and other significant findings | N/A |
|  |  |
| Physical Exam - List exam maneuvers expected for this case and any abnormal findings that SP will simulate. (tenderness, hyper-hypo reflex, rebound, weakness, etc.)  None | |
| PHYSICAL EXAM FINDINGS |  |
| 1. Written in layperson’s terms | N/A |
| 1. General appearance - affect, appearance, position of patient at opening (i.e., sitting, lying down, holding abdomen, etc.) | Sitting, irritable affect |
| 1. Vital signs | HR 80; BP 135/86 RR: 18. |
| 1. Specific findings and affect | Irritable |
| 1. Response to certain physical movements | N/A |
|  |  |
| DIAGNOSIS AND DIFFERENTIAL |  |
| Diagnosis with support from positive and negative history and PE findings | No diagnosis |
| Differential with support from positive and negative history and PE findings | None |
|  |  |
| MANAGEMENT OR DIAGNOSTIC PLAN | Purpose is not to fix the patient’s problem, but to verbally de-escalate. Student may offer options for sleep hygiene. |
|  |  |
| PROFESSIONALISM ISSUES OR CHALLENGES | Challenge is to verbally de-escalate the patient and not offer xanax. |
